# Strengthening Health Systems at Facility-Level: Feasibility of Integrating Antiretroviral Therapy into Primary Health Care Services in Lusaka, Zambia

**DOI:** 10.1371/journal.pone.0011522

**Published:** 2010-07-13

**Authors:** Stephanie M. Topp, Julien M. Chipukuma, Mark Giganti, Linah K. Mwango, Like M. Chiko, Bushimbwa Tambatamba-Chapula, Chibesa S. Wamulume, Stewart Reid

**Affiliations:** 1 Centre for Infectious Disease Research in Zambia, Lusaka, Zambia; 2 Schools of Medicine and Public Health, University of Alabama, Birmingham, Alabama, United States of America; 3 Lusaka District Health Management Team, Lusaka, Zambia; 4 Ministry of Health, Lusaka, Zambia; University of Cape Town, South Africa

## Abstract

**Introduction:**

HIV care and treatment services are primarily delivered in vertical antiretroviral (ART) clinics in sub-Saharan Africa but there have been concerns over the impact on existing primary health care services. This paper presents results from a feasibility study of a fully integrated model of HIV and non-HIV outpatient services in two urban Lusaka clinics.

**Methods:**

Integration involved three key modifications: i) amalgamation of space and patient flow; ii) standardization of medical records and iii) introduction of routine provider initiated testing and counseling (PITC). Assessment of feasibility included monitoring rates of HIV case-finding and referral to care, measuring median waiting and consultation times and assessing adherence to clinical care protocols for HIV and non-HIV outpatients. Qualitative data on patient/provider perceptions was also collected.

**Findings:**

Provider and patient interviews at both sites indicated broad acceptability of the model and highlighted a perceived reduction in stigma associated with integrated HIV services. Over six months in Clinic 1, PITC was provided to 2760 patients; 1485 (53%) accepted testing, 192 (13%) were HIV positive and 80 (42%) enrolled. Median OPD patient-provider contact time increased 55% (6.9 vs. 10.7 minutes; p<0.001) and decreased 1% for ART patients (27.9 vs. 27.7 minutes; p = 0.94). Median waiting times increased by 36 (p<0.001) and 23 minutes (p<0.001) for ART and OPD patients respectively. In Clinic 2, PITC was offered to 1510 patients, with 882 (58%) accepting testing, 208 (24%) HIV positive and 121 (58%) enrolled. Median OPD patient-provider contact time increased 110% (6.1 vs. 12.8 minutes; p<0.001) and decreased for ART patients by 23% (23 vs. 17.7 minutes; p<0.001). Median waiting times increased by 47 (p<0.001) and 34 minutes (p<0.001) for ART and OPD patients, respectively.

**Conclusions:**

Integrating vertical ART and OPD services is feasible in the low-resource and high HIV-prevalence setting of Lusaka, Zambia. Integration enabled shared use of space and staffing that resulted in increased HIV case finding, a reduction in stigma associated with vertical ART services but resulted in an overall increase in patient waiting times. Further research is urgently required to assess long-term clinical outcomes and cost effectiveness in order to evaluate scalability and generalizability.

## Introduction

In Zambia as elsewhere in sub-Saharan Africa, HIV care and treatment is primarily delivered in stand-alone or vertical antiretroviral (ART) clinics located next to primary health care clinics [1], [2], [3].Vertical HIV services have helped fulfill the mandate of emergency scale-up set by the WHO 3x5 initiative to rapidly enroll large numbers of HIV-infected patients by permitting implementers to bypass existing public health systems, set up parallel logistical and service-delivery arrangements and concentrate on intensively training select staff [4]. Recent evidence demonstrates that the establishment of vertical HIV services in high-prevalence settings has catalyzed the refurbishing of laboratories and clinics, strengthened management systems for supply chains, and improved training for professional and lay health care workers [5], [6]. However, some have suggested that this approach has further weakened the national health system [7], [8], [9] and the continued separation of ART clinics from other primary health departments raises questions relating to sustainability of HIV care and treatment, distribution of human resources, access and equity of care, space and infrastructure availability, continuum and quality of care, and stigma. Recognizing emergent concerns related to the middle- and long-term impact of vertical HIV service delivery, the Lusaka District Health Management Team (LDHMT) initiated efforts in July 2007 to develop a model of fully integrated ART and regular non-HIV outpatient department (OPD) services to pilot in four urban clinics in Lusaka District. This paper presents results from a feasibility assessment based on the first two clinics.

### Intervention

To develop a model of integrated service delivery, the Centre for Infectious Disease Research in Zambia (CIDRZ) working with LDHMT, conducted site assessments of the ART and OPDs of ten urban primary health care clinics, documenting and comparing clinical space, staff availability, referral protocols, patient flow and medical record keeping. Findings were analyzed and cross referenced to provide the basis for a model of integrated service delivery which focuses on three key modifications: i) amalgamation of physical space and patient flow; ii) standardization of medical records and screening forms and iii) introduction of routine provider initiated testing and counseling (PITC).

**Figure 1:**
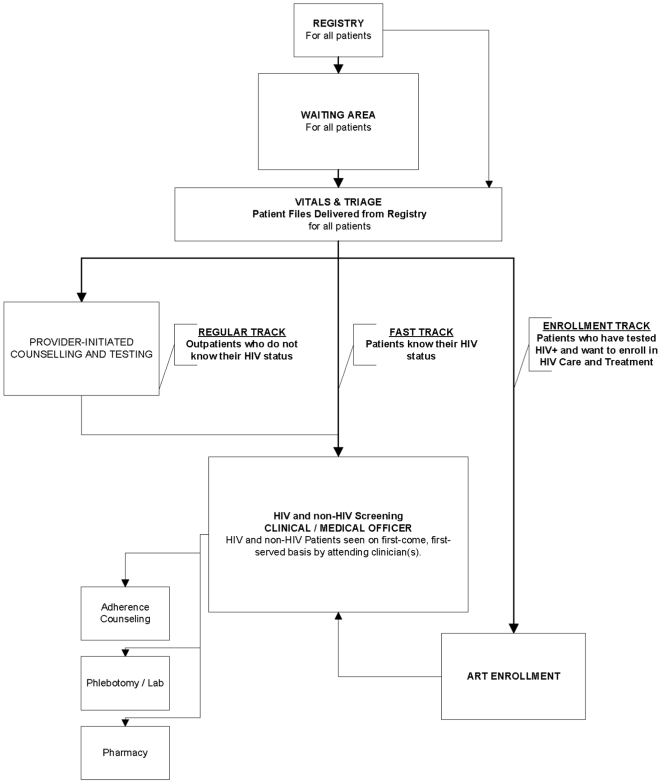
Integrated patient flow.

Primary operational objectives of the pilot were to i) increase HIV case finding; ii) improve adherence to clinical care protocol for OPD patients iii) maintain adherence to clinical care protocol for ART patients; iii) reduce clinic-based HIV-associated stigma and iv) improved continuum of care between departments. To meet these objectives, the integrated patient flow adopted a modified first-come, first-serve approach with a fast-track mechanism (Fig. 1). All patients attended a single clinic registry and had their medical files delivered to a single vitals station. Patients queued to have their vital signs recorded by a nurse and/or lay health worker and were triaged to one of three ‘tracks’.

Fast Track was for patients who knew their HIV status, including those already enrolled in ART or those who had received a HIV test within 6 months. Fast Track patients moved directly from vitals to screening by a clinician, after which they could visit a range of services depending on their needs (Fig. 1). Regular Track was for patients who did *not* know their HIV status. Any patient who did not have a recent (<6 months) written record of a negative HIV test was offered counselling and opt-out (finger-prick) testing by one of two lay counsellors with results recorded in patient files. This is an innovation in Zambia, where routine PITC is not offered in OPDs, and patients must attend stand-alone VCT services in order to test. Patients testing positive were offered a chance to enrol in the ART program immediately or return at a later (pre-determined) date. Finally, Enrolment Track was for returning HIV positive patients referred for ART enrolment. After recording of vitals signs, patients in this track went through enrolment/registration procedures before being screened by a clinician. On subsequent visits, these patients were triaged to Fast Track (Fig. 1). Pharmacy and laboratory services were integrated at point-of-delivery, such that OPD and ART patients were seen by the same health care worker(s), at the same station, on a first-come first-serve basis. Pharmacy store rooms were combined, however, requisition of ART drugs and supplies continued to be separate from OPD requisitions, based on current national systems. Similarly, processing of blood tests for ART patients remains offsite while non-HIV lab tests (e.g. malaria or tuberculosis) are processed at the clinic or the closest reference clinic.

In the vertical system, ART staff are part of an existing pool of Ministry staff including nurses, clinical officers (COs), doctors (MOs) and lay personnel. Those who have ART training are eligible to work in the ART clinics and receive ‘over-time’ payment for this work [10]. In this integrated pilot, staff without ART experience received ART training either prior to or within three months of integration. All health care providers from the two departments were then combined to form a single cadre dealing concurrently with OPD and ART patients. These included lay health care workers previously trained and assigned to work only in ART. Free-text notebooks used in vertical OPD departments were replaced with folder-files to match ART patients' making medical records visually indistinguishable. Unique computer-generated patient numbers initially developed for use in ART program were allocated to all existing and new OPD patients. Separate ART and OPD patient ID cards were replaced with a uniform patient ID card. A standardized OPD screening form was also developed incorporating a series of prompts to record patients' vital signs and HIV test results, conduct symptom checks, and screen for opportunistic and other infections.

### Clinic Selection & Implementation

Clinics 1 and 2 were purposively selected by District officials based on moderate catchment population (25,000 and 60,000 respectively), presumed high rates of undiagnosed HIV, and extreme space and staffing constraints that were being exacerbated by vertical departments. In the first phase of implementation, a series of information and planning meetings were held with clinic staff and community representatives. Initial meetings involved working with clinic leadership to map available infrastructure and plan re-allocation of space to accommodate the integrated patient flow. Essential small-scale renovations were planned such as installation of shelving to accommodate files. Meetings with the wider clinic staff were held to introduce the concept of integration and the specific model being implemented, providing a chance for staff to voice concerns and offer feedback. Meetings with neighbourhood health committees (NHC) also took place to inform members of the upcoming changes and provide them with information to disseminate to their respective catchment areas.

During the second phase, community sensitization was scaled up to include drama performances by a trained theatre troupe in strategic locations. Performances were designed to use common themes and experiences to inform community members that clinic services would be integrated and to explain how the changes would affect them. Renovations were completed and a series of three didactic and interactive staff trainings were conducted with lay and professional health workers to ensure familiarity with the integrated model. In the third phase clinic space was re-arranged to accommodate the altered patient flow and the integrated service model initiated. For three weeks following initiation, intensive support and in-service mentoring for clinical and registry staff was provided to facilitate a smooth transition. Thereafter on-going monthly follow-ups were conducted by CIDRZ staff with LDHMT oversight.

## Methods

### Ethics Approval

University of Alabama IRB: Protocol No: X080403013; Continuing Review received 28 April 2009

University of Zambia REC: Protocol No: 003-02-08; Continuing Review received 28 April 2009

This feasibility study collected data in six areas matching the pilot objectives. Rates of clinic-based HIV case-finding and referral; adherence to collection of vital signs for ART and OPD patients; adherence to 6 indicators of clinical protocol for ART patients; amount of with-provider time and per visit waiting times for OPD and ART patients and finally, patient and provider perceptions.

HIV case-finding and referrals were measured by collating operational data from clinic registers on routine PITC services (Appendix S1, S2) as well as pre-existing VCT services. Indicators included the number of patients counseled, number accepting testing, number HIV-positive and number actually enrolled in ART. All patients accessing PITC and VCT six months pre- and post-implementation were captured. No personal or otherwise identifying information was recorded.

Adherence to collection of patient's vital signs was assessed via random file review of 100 OPD and ART files at each site. This provided an estimate of the number of patients with vital signs recorded at their last visit, pre- versus post-implementation. Adherence to 6 indicators of ART protocol was measured for all patients enrolled in the ART program at both sites, during the two quarters pre and two quarters post integration. Data for the two sites were extracted from the electronic medical record system on a quarterly basis starting from April 2008 through July 2009. Indicators were selected to investigate associations (not measure causation) and included: (1) percentage of newly enrolled patients with a baseline CD4 measurement, (2) percentage of delinquent patients per quarter (3) percentage of newly enrolled, eligible patients who are on antiretroviral drugs (4) percentage of newly enrolled, eligible patients who are on cotrimoxizole, (5) percentage of newly enrolled patients on zidovudine who had a hemoglobin measurement, (6) percentage of active patients who attended a scheduled follow-up visits in the last four months. A two-sided Wilcoxon Rank Sum Test was used to measure differences in performance between: i) the quarter immediately pre- and quarter immediately post-implementation and ii) the quarter immediately pre- and the second quarter post-implementation.

A time-in-motion study was conducted to measure median waiting and with-provider times per patient visit pre- and post-implementation. With-provider and per-visit waiting times were intended to provide a rough proxy for quality of care and provide a standard indicator that could be used to compare the effect of integration across the two cadres. Data were collected over two seven day periods, pre- and post-implementation. Data was recorded using a study form (Appendix S3, S4) attached to the medical file of every patient arriving at the clinic prior to 12.00. All patients visiting the clinic(s) during the seven day period were captured. Study staff based in the registry recorded the time of patient-arrival and type of patient (OPD or ART) visit on study forms. Using a synchronized clock at each clinical station, (vitals, triage, screening room, laboratory, pharmacy, adherence, ART enrolment), the start and finish times for each patient interaction was recorded on the form by the attending health care worker. Time of ‘exit’ was taken as the finish time noted by the last attending provider. At the end of a patient's visit, study forms were removed from the medical file and data was manually entered into Excel spreadsheets and imported into SAS version 9.1.3 (Cary, NC, USA) to analyze median total and intra-station waiting times as well as consultation times for each clinical station. No personal or otherwise identifying information was recorded. Identical studies were conducted pre- and post-integration, with two-sided Wilcoxon Rank Sum Tests used to compare differences in medians.

Patient and provider perceptions were collected in one-on-one interviews using semi-structured questionnaires and free-listing techniques. Patient interviews investigated perceptions of their experience in either the vertical or the integrated services asking them to list positive and negative features of their visits. Patients were randomly selected and interviews were conducted in a private room by trained community interviewers in English, Nyanja or Bemba. Interviews with health care workers were conducted in English, and focused on positive and negative aspects of working in the clinic, pre- and post- integration. Interview questions were open ended and responses were manually recorded, entered into an electronic database, coded inductively and analyzed for common themes. All participants provided written informed consent.

This study was approved by the University of Alabama at Birmingham Institutional Review Board (Birmingham, AL, USA) and the University of Zambia Research Ethics Committee (Lusaka, Zambia).

## Results

### HIV Case Finding and Referral

**Figure 2:**
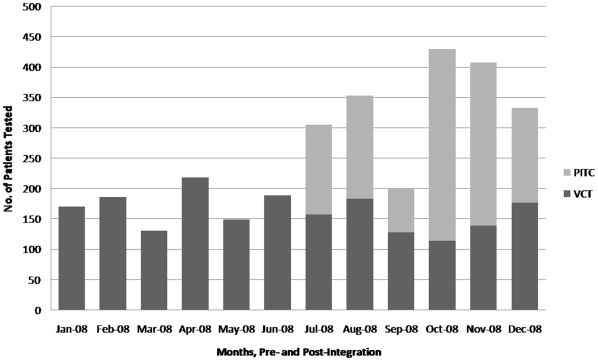
Clinic 1 testing pre- and post-integration.

**Figure 3:**
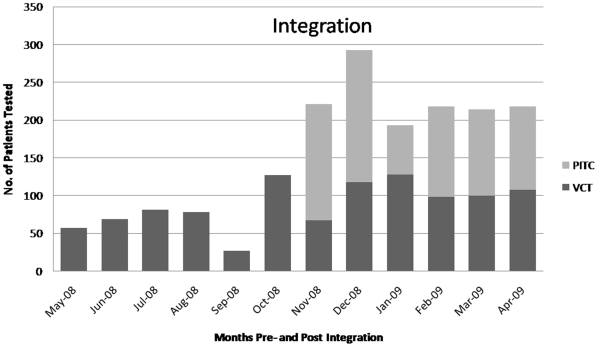
Clinic 2 testing pre- and post-integration.

In Clinic 1 over six months, PITC was provided to 2760 OPD patients, with 1485 (53%) accepting testing, 192 (13%) found HIV positive and 80 (42%) subsequently enrolled in ART care with a minimum follow up of 6 months. In six months of integration at Clinic 2, PITC was offered to 1510 OPD patients, with 882 (58%) accepting testing, 208 (24%) HIV positive and 121 (58%) enrolled in care. Although it is beyond the scope of this particular paper to investigate characteristics of those enrolled in ART, continued demand for clinics' stand-alone voluntary counseling and testing (VCT) service suggests that the population being reached by PITC is different from that accessing VCT (Fig. 2,3).

### Collection of Vitals

From a pre-implementation baseline of nil, vital signs were collected during 76% (n = 76) and 73% (n = 73) of OPD patients' latest visits at Clinic 1 and 2 respectively. No change was observed in collection of vitals for ART patients with over 96% recorded pre- and post-integration.

### Time-in-motion

**Figure 4:**
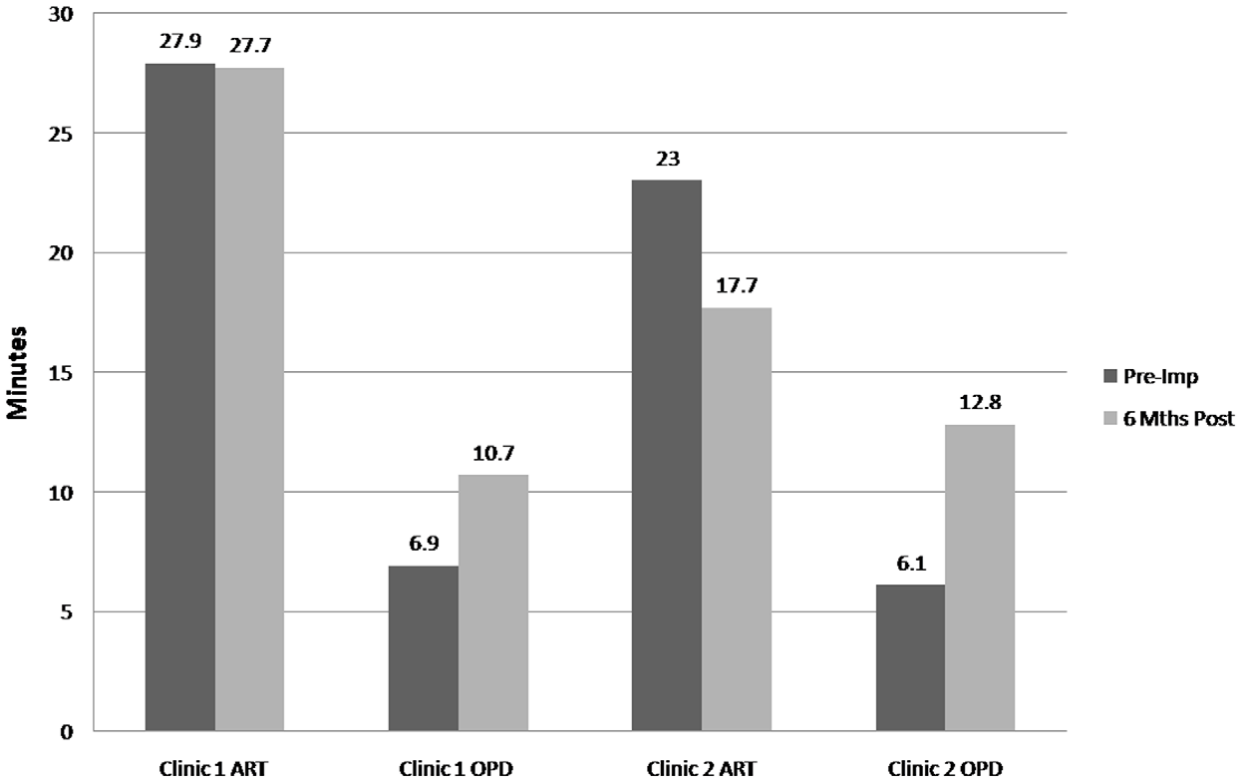
Median time spent with any health care worker.

**Figure 5:**
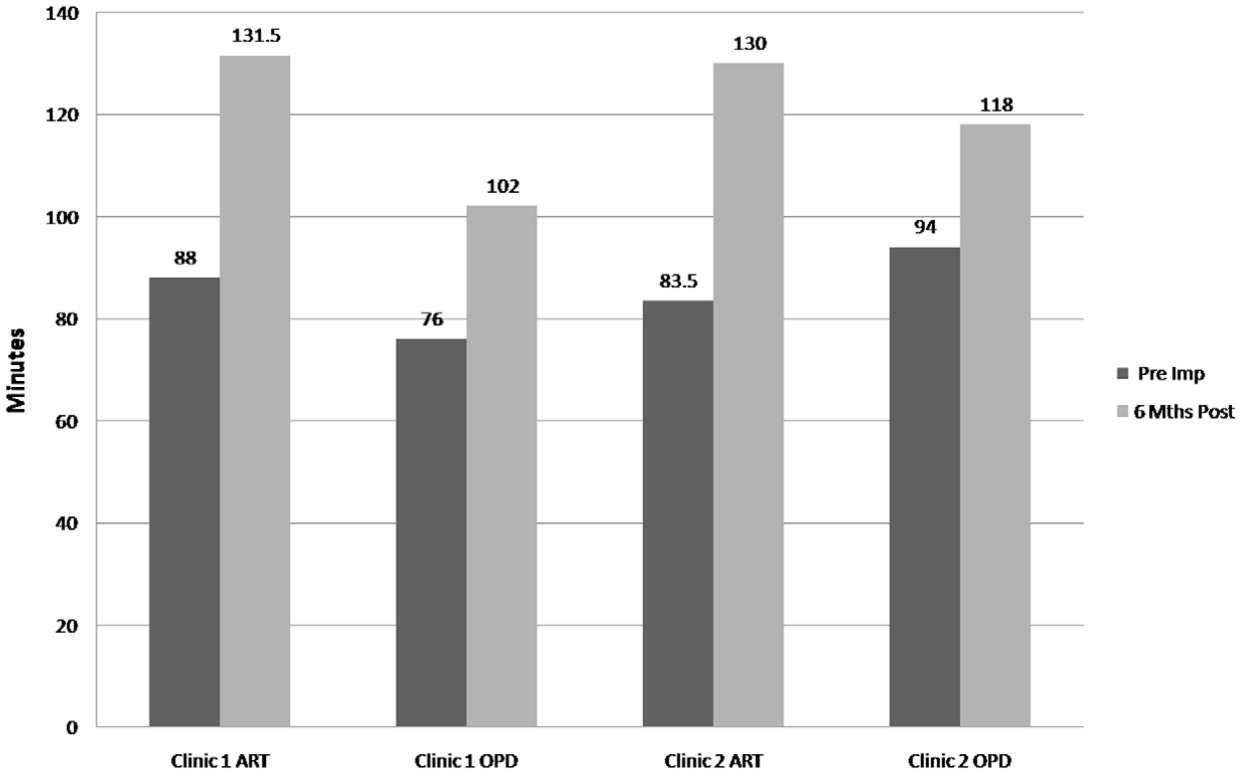
Median waiting times per visit in ART and OPD.

In Clinic 1, data on 151 ART and 357 OPD patients were captured in the pre-implementation time-in-motion study, and data on 129 ART and 385 OPD patients post-implementation. Six month follow-up at Clinic 1 showed 55% increase (6.9 vs. 10.7 minutes; p<0.001) in median patient-provider contact time per OPD visit and 1% decrease (27.9 vs. 27.7 minutes; p = 0.94) for ART patients (Fig. 4). Median time spent with a clinical officer or doctor per visit remained virtually unchanged for ART patients (10.1 vs. 10.8 minutes; p = 0.45) while there was some increase for OPD patients (4.1 vs. 5.2 minutes; p<0.001). Median waiting times increased by 43 (p<0.001) and 26 minutes (p<0.001) for ART and OPD patients, respectively (Fig. 5).

In Clinic 2, data on 201 ART and 585 OPD patients were captured pre-implementation, and on 221 ART and 248 OPD patients post implementation. In both clinics, seasonal fluxes in weekly and monthly clinic attendances are common and may account for the uneven OPD attendance pre- and post-implementation (Appendix S5 demonstrates shifts in monthly clinic attendance at Clinic 1 and 2 between 2006–2008). Six-month follow-up at Clinic 2 demonstrated a 110% increase in median OPD patient-provider contact time (6.1 vs. 12.8 minutes; p<0.001) and a 23% decrease (23 vs. 17.7 minutes; p<0.001) for ART patient-provider contact (Fig. 4). Median time with a clinical officer/doctor saw a slight decline for ART patients (10.3 vs. 9.6 minutes; p = 0.51) while there was a small but significant increase for OPD patients (4.0 vs. 5.8 minutes; p<0.001). Median waiting times increased by 46 (p<0.001) and 24 minutes (p<0.001) for ART and OPD patients, respectively (Fig. 5).

### Provider Adherence to ART Clinical Protocol

**Figure 6:**
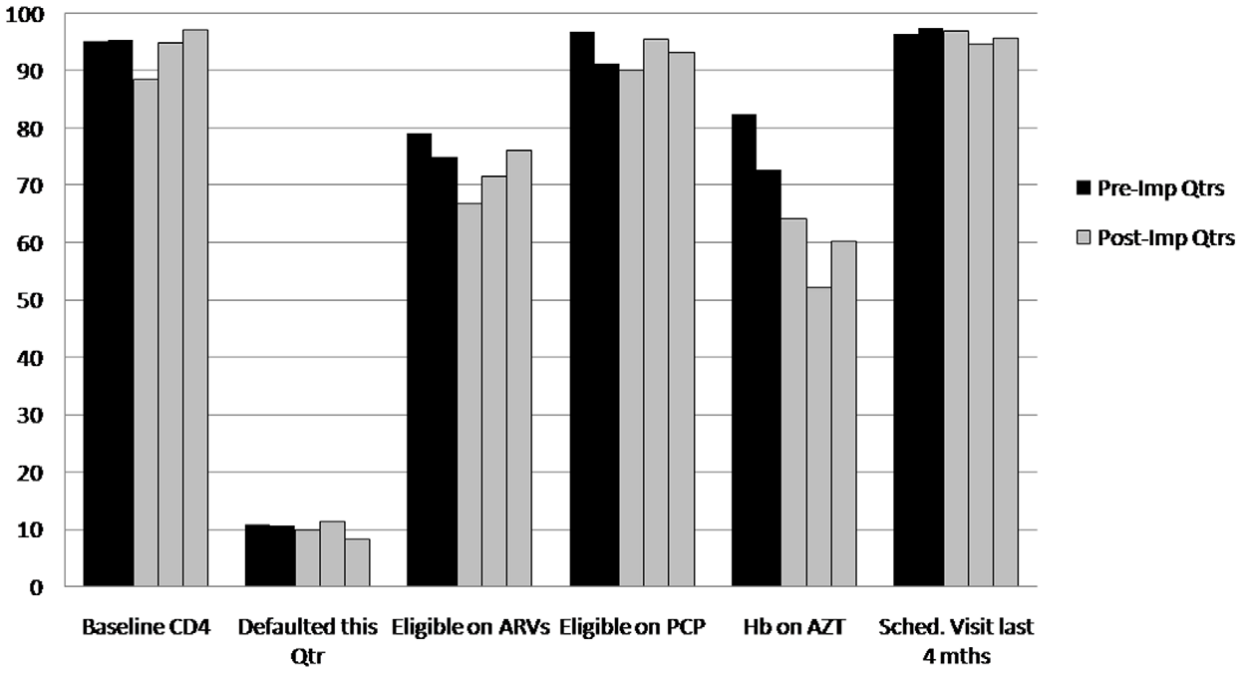
Clinic 1 ART quality assurance indicators.

**Figure 7:**
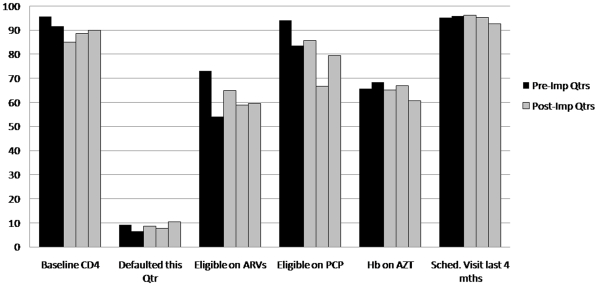
Clinic 2 ART quality assurance indicators.

Figures 6 and 7 summarize quarterly measurements for six indicators of adherence to ART protocol. In Clinic 1, the percentage of patients with baseline CD4 collected decreased from pre- to first quarter post-implementation (95.3%–88.5%, p = 0.01) but subsequently recovered with no difference between pre- and two-quarters post-implementation (95.3%–97%, p = 0.35). A decline in the percentage of patients with hemoglobin measured while on zidovudine was observed between pre- and two-quarters post-implementation (72.7%–60.3%, p = 0.02). For all other indicators in Clinic 1, no difference was measured. In Clinic 2, an increase in the percentage of delinquent patients was observed between pre- and first quarter post-implementation (6.6%–8.7%, p = 0.04), as well as pre- and second quarter post-implementation (6.6%–10.5%, p = 0.003). This is partially explained by the small numbers involved, as well as the unusually low rate of delinquency in the quarter preceding implementation. There was also a difference in percentage of patients with hemoglobin measured while on zidovudine between pre- and second quarter post-implementation (68.4%–60.6%, p = 0.04); no other indicators demonstrated significant change.

### Patient Perceptions

Semi-structured interviews were carried out with ART and OPD patients in Clinic 1 (Post: n = 16) and Clinic 2 (Pre: n = 16; Post: n = 17). Table 1 and 2 summarizes common perceptions pre- and post-implementation. In both pre- and post- interviews, a majority of respondents at both sites classified health providers as ‘helpful’ ‘supportive’ and/or ‘caring’ while common concerns about health services included long waiting times, insufficient staff, and drug and/or equipment shortages. More than half the respondents (post- at Clinic 1, and pre- and post- at Clinic 2) expressed a negative view of vertical ART and OPD services, with the most common reason being that separation contributed to stigma. For patients who expressed a positive view of vertical services, the most common reason was that ART patients felt more comfortable in the presence of other HIV-clinic patients or ‘could be free amongst themselves’. More than half the respondents (post- at Clinic 1, and pre- and post- at Clinic 2) expressed a positive view of integrated or combined services. The two most common reasons given were that combined services would improve equity between OPD and ART and reduce stigma associated with accessing HIV treatment. Negative views of integration stemmed from two major concerns. The first focused on the reduced opportunity to discuss issues related to HIV and share coping mechanisms with fellow ART patients. Whereas in the vertical system ART patients said they were ‘comfortable’ a number expressed concerns about confidentiality and ‘not feeling free’ in the integrated service. The second concern was that integration increased waiting times.

**Table 1:** Pre- and post-implementation patient perceptions.

PATIENT: PRE Concerns with Clinic Operations	PATIENT: POST Concerns with Clinic Operations
*Staff are supportive, but too slow, maybe it is because the clinic is small or it is because they are used to see patients every day [Clinic 2]*	*It's bad when sometimes you go home very late and sometimes no medicine [Clinic 2]*
*Staff are not enough, [Clinic 2] is a small clinic patients are many and few staffs so when they are tired they become frustrated to patients. [Clinic 2]*	*You will be delayed for so many hours and at the end you will find that there is no drugs [Clinic 2]*
*There are long queues waiting for treatment [Clinic 1]*	*Long queue, they take too long to attend to you [Clinic 1]*
**Perceptions of Separated OPD & ART**	**Perceptions of Separated OPD & ART**
*It's very okay… because patients from ART clinic know themselves no one will break the news in the community. [Patient, Clinic 2]*	*Before integration those patients belong to ART we knew ourselves and we knew we have come for ARVs so no one will go and stigmatize [Clinic 2]*
*The system is bad because patients feel bad and they even stigmatize themselves thinking that those with HIV are more dangerous than those who are attending the OPD. [Clinic 2].*	*The separation was good, because us who were at ART clinic we used to encourage one another…but these days it is difficult to share our problems freely [Clinic 2]*
*The separation is okay because we feel free to discuss our HIV status among ourselves because we know that we are all positive. [Clinic 2]*	*Separation is not a good thing. That is a reason there was stigma …they were scared because of the isolation of OPD and ART [Clinic 2]*
*It is not right reason being that people in ART receive drugs all they need an like those in OPD sometime they run out of drugs and its brings tension in the patients [Clinic 2]*	**Perceptions of Integrated OPD & ART**
**Perceptions of Integrated OPD & ART**	*These days staff treat patients very well, but in the past patients that were being treated good were only those who are at ART clinic” [Clinic 1].*
*This [idea] is a very confusing thing, because many people are going to be infected. Patients who are positive and they are very sick and TB patients coughs too much and very carelessly. [Clinic 2]*	*It is a good thing, because some of us it is when we even decided to test for HIV very freely, in sense that no one will know if I am positive or not [Clinic1]*
*I think it is a good idea because it is going to help a lot of people to go to the clinic, its because people will not stigmatize each other.[Clinic 2]*	*These days they check your weight, temperature BP and even test you for HIV if you want [Clinic 1].*
*It's not a good idea cause we are not going to be free, the way we interact with each other, sharing problems it is not going to be easy… [Clinic 2]*	*…they have combined the two clinics and that is good, so next week Monday I will come for [testing] since no-one will know [Clinic 2].*
*It is good because they will be no segregation among the patients themselves [Clinic 2]*	*We are using files instead of books, and pink cards. It's a good idea because every patient is using what the other patient is using [Clinic 2].*

**Table 2:** Pre- and post-implementation provider perceptions.

PROVIDER: PRE Positive Aspects of Work	PROVIDER: POST Positive Aspects of Work
*It's so interesting because I like working with patients, children and pregnant mothers [Clinic 2]*	*We have more knowledge now whereby we have come to know that this patient has come for pharmacy or for lab [Clinic 2].*
*I feel good because I work to help people especially those not feeling good who are ill. [Clinic2]*	*It is a good thing to interact with different patients with different illnesses [Clinic 2].*
*I feel motivated because whenever I do come, there are a lot of things, challenges, things new to learn and the knowledge is increasing [Clinic 1]*	*I feel very good, because I have seen that [integration] is going to help other people to be free when coming to the clinic [Clinic 1]*
**Negative Aspects of Work**	**Negative Aspects of Work**
*Of late is quite busy, due to lack of CO and we even help them to screen patients which makes us tiresome. [Clinic 2]*	*Patients complain a lot if you are slow or you make them overstay in the clinic. [Clinic 2]*
*The bad thing is only when we run out of things we need…such as needles and syringes. And understaffing; more patients than nurses, so as a result, sometimes we do things very fast that maybe the patients didn't even understand [Clinic2]*	*It's average, especially those on ART we can't reveal we know them and we just pull their files and let them go to adherence even those from OPD when they go through DCT We don't reveal their status to anybody else [Clinic 2].*
*There is a lot of work which I can call it overload. [Clinic 1]*	*Tiresome, we are few staff so there is work overload compared to the staff. [Clinic 1]*
*Understaffing. You are one person doing everything, doctor, nurse, dispenser sometimes even cleaner, so maybe you have to cater for all those things [Clinic 2].*	**Differences between OPD and ART**
**Differences between OPD and ART**	*Nowadays there is no division that this work is for OPD or ART. Since the integration everyone is working together since we are one [Clinic 2].*
*You notice something about this clinic – it's special; [providers are] very conscious in how they interact with their patients…There isn't a shortfall of anything in ART but in OPD you don't have maybe thermometers, BP machines. [Clinic 1]*	*We are learning a lot and have created a good relationship between ourselves from OPD and ART [Clinic 2]*
*ART nurses are really doing nursing care… compared to OPD where you have one hundred [patients] in the morning and only 2 thermometers [Clinic 1,]*	*I feel good, because this integration has let us to be united (one). No OPD staff nor ART staff. [Clinic 1]*
*Most of the time towards work in OPD depends on the individual, [but] the set up in ART is systematic [Clinic 1].*	*A good thing is that most of the workers are united [Clinic 1].*
*Most nurses run from OPD to work at ART because they know that they are getting some incentives [Clinic 2].*	*We are able to know how to keep records especially for ART patients. Since the integration now I have come to know everything…It's good we are working together [Clinic 2].*

### Health Provider Perceptions

Semi-structured interviews were conducted with ART and OPD staff in Clinic 1 (Pre: n = 13; Post: n = 16) and Clinic 2 (Pre: n = 15; Post: n = 16) summarized in Table 1 and 2. Pre-implementation interviews identified considerable tension between staff working in ART and OPD. Common reasons included the opportunity for ART nurses to earn more; ART nurses acting in a superior manner to their OPD colleagues; segregation and lack of communication between OPD and ART staff and more rigorous procedures and/or better quality assurance procedures in ART. Other negative aspects of work identified in both departments during pre-implementation interviews included drug and/or equipments shortages, personal fatigue and overwork. At both sites positive aspects of pre-implementation work focused on helping patients and providing a service. Post-implementation, three new positive aspects of clinic work were identified at both sites: working together with no divisions, helping more HIV-infected people test and enroll and a reduction in stigma. Negative perceptions of integrated services amongst the professional health care workers focused on perceived increase in work load. This was particularly strong amongst professional health workers at Clinic 1. Despite similar shifts in work environment, this complaint was rarely expressed by lay health care workers at either site.

## Discussion

In this report, we demonstrate the feasibility of complete integration of HIV care and treatment with non-HIV outpatient services in a high-HIV prevalence, low-resource setting. Over the first six months, positive outcomes matching the pilot objectives included: i) an additional 4270 patients being counseled of whom 2367 (55%) accepted testing, 400 (17%) identified as HIV-positive, and 201 (50%) enrolled into HIV care; ii) A 70% increase (from a baseline of zero) on OPD patients receiving pre-screening collection of vital signs; iii) a reduction in both patient and staff perceptions of stigma associated with HIV care and treatment; iv) improved staff communication and teamwork. Negative or unintended outcomes of the pilot included an increase in the waiting times for both OPD and ART patients and a drop in some indicators for delivery of care for ART patients; a further concerning outcome was some ART patients' perception that the integrated service provides a less secure environment in which to share experiences with fellow patients.

Although small in scale, this intervention constitutes perhaps the first documented attempt to fully integrate ART and OPD services in a setting where HIV care and treatment was established vertically. This pilot stands apart from other documented integration models in that it constitutes a complete harmonization of point-of-care HIV and non-HIV services versus strengthened referral between still vertical services [11], [12] or decentralization of ART from tertiary to primary care settings [13], [14]. It also presents a full account of implementation and service delivery arrangements including descriptions of where and how care is provided, what information and technology systems are utilized and critically, how the systems were monitored. In doing so, this paper is a first step in addressing the call for evidence of feasible approaches to point-of-care health systems strengthening, using a model of integrated HIV and primary health care [15], [16]. The following sections describe three ways in which this model enabled us to strengthen both supply and demand side factors affecting primary health care service delivery [17], [18], [19].

### HIV Case Finding and Stigma

Over time, the stand-alone nature of both HIV testing and HIV treatment services has contributed to a culture of HIV/AIDS exceptionalism in the Zambian health care system. Patients independently seeking testing or treatment may be deterred by the stigma associated with being seen at that service. Patients *recommended* for testing by a clinician are required to visit stand-alone services before returning to the clinician with their result. The stigma associated with being seen at VCT, fear of the test itself and lack of accompaniment by clinic staff have been shown to affect testing uptake and reduce the likelihood of HIV-positive patients returning to seek care [20]. Structural barriers including distance to the clinic, transport costs and lost work hours make the burden of additional queuing and the prospect of future visits even less attractive.

With a national HIV prevalence of 14.3% and 50% of HIV- infected individuals not yet in care[21], [22] new approaches to strengthening uptake of testing and referral to care and treatment in Zambia are urgently needed [23], [24]. This integrated model employed three strategies to address the atmosphere of exceptionalism that may be undermining access to HIV testing treatment services [25], [26], [27]. First, by introducing routine PITC the model sought to minimize barriers to testing by making it a routine service provided in a non-stigmatizing environment. Second, with the standardization of patient ID and medical files this model sought to minimize the chance of ART patients being identified by fellow patients in the clinic, thereby reducing the stigma associated with receiving HIV care and treatment. Finally, the unified patient flow encouraged health care workers themselves to adopt a non-segregated approach to delivery of care.

### Leveraging Human Resources & Space

Vertical ART and OPD services have exacerbated already limited space and human resources in the Zambian health care setting. WHO recommends a minimum of 20 physicians and 100 nurses per 100,000 population while in 2007, Zambia had with 7 physicians, 9.2 clinical officers and 113 nurses per 100,000 spread across *all* health services[28] With the prospect of a certain rise in the number accessing chronic, life-long HIV care, an even larger burden on material and human resources for health can be anticipated [6]. Integration of OPD and ART care removed the need for dual registries, pharmacies and other duty stations freeing valuable space and staff time for pre-screening, screening and routine PITC. Combining the OPD and ART workforce facilitated nursing duty rosters that reduced the level of multitasking previously required. Clinicians receive OPD and ART patients on a first-come first-served basis instead of doing back-to-back shifts in different departments. Task shifting to lay cadres (most commonly found in ART clinics) resulted in benefits to both ART and OPD patients; for example lay counselors in the integrated model provide health education talks to all patients, not just ART patients. Time-in-motion data demonstrated that with the same level of human resources, the integrated model enabled more time to be spent with OPD patients while consultation times with ART patients were maintained or modestly reduced. Harmonization of staff rosters also eliminated the opportunity for staff to ‘double-claim’ by signing up for an ART overtime shift while already rostered for regular OPD duty.

### Clinic Systems

Pre-implementation interviews demonstrated that vertical services contributed to ART and OPD staff working in relative isolation hindering communication, transfer of medical records and referrals. Continuum and quality of care was undermined by logistical breakdowns that diminished clinicians' ability to make accurate diagnoses, prescribe appropriate medications, and track potential drug interactions. The well documented difficulties involved in referring patients between vertical OPD, ART, antenatal care and other services were similarly experienced in this setting [28]. Post-implementation provider interviews demonstrate that integrating OPD and ART services helped staff to work as a team, improving communication and eliminating the opportunity to attribute failure to meet a patient's needs to another department. Referrals ‘between’ OPD and ART were immediate and in situ simplifying the logistics of patient movement. Unified record keeping and filing systems ensured that patients could not have separate OPD and ART files, reducing the chance of inappropriate care due to incomplete medical records. In addition, standardized OPD screening forms and the recording of vital signs at every visit contributed to a more consistent and comprehensive approach to OPD care. Nonetheless, monitoring of quarterly ART performance reports identified a decline in adherence to some ART clinical care protocols suggesting careful monitoring and ongoing mentoring will be necessary to maintain standards established at stand-alone clinics.

### Operational Challenges & Lessons Learnt

Results from this study indicate a number of operational challenges. First, this model of integrated HIV and primary health services resulted in increased patient waiting times. Disaggregated results from the time-in-motion study (not shown) suggest that the increase is attributable to two factors. First, the re-introduction of collection of OPD vital signs and the addition of PITC automatically increased patient waiting time by creating additional stops in the patient flow. Second, by combining two groups of patients and increasing the number patients moving through a single system, small delays at the *beginning* of the patient flow (e.g. registry or vitals) resulted in exponentially larger delays further along the process. For example, a 15–20 minute delay in transferring the first files from registry to vitals can result in the final ‘station’ (typically pharmacy) not seeing a patient for several hours.

Although visit times remained within manageable limits in the pilot sites literature documenting the effects of waiting times clearly indicate that longer wait times can be a barrier to retention in care [29], [30]. A work culture of timeliness is thus more critical in an integrated system where queues are necessarily longer. We conclude that training and supportive supervision to address this issue should be included in the formative stages of the intervention. Additionally, modeling this data to simulate the most efficient distribution of available human resources in clinics with larger or smaller patient populations may be useful in prioritizing integration for certain facilities and tailoring the approach to reduce bottlenecks and attendant waiting times.

Routinely collected data on HIV case-finding and referral demonstrated significant increases in the uptake of testing but only a minimal increase in ART enrollment rates with six month follow-up. Since delay between the time-of-testing to time-of-enrolment is common, a longer follow-up period may demonstrate overall higher rates of enrolment. However, these results highlight the continued difficulty in enrolling HIV patients into care and we posit that an integrated service may facilitate increased HIV case-finding but not improve the rates of successful enrolment into care. Further work to systematize this process and promote duty-based responsibilities is necessary to minimize reliance on individuals.

Post-integration patient interviews indicated that many OPD and ART patients experienced a reduction in perceived stigma associated with HIV care and treatment in the integrated service. A contradictory finding was that some ART patients felt *less* able to discuss their problems with fellow patients due to the mixing of OPD and ART queues. Careful consideration of these two factors is necessary when planning integration. Extensive community sensitization in advance of integration minimizes the shock experienced by patients attending a newly integrated clinic, while ongoing, within-clinic patient sensitization and counseling is essential to strengthen understanding and a sense of security among both cadres of patients.

Although this study did not formally evaluate infrastructure, we note that appropriate infrastructure is an essential pre-requisite to effective integration. Inclusion of provider-initiated counselling and testing and harmonized registry and pharmacy services all require re-allocation of space. In this setting, innovative and experimental approaches were necessary to make use of limited space while ensuring appropriate infection control measures were in place. Nonetheless, in some sites integration of this type would not be possible without significant renovations or new infrastructure altogether.

### Limitations

This pilot was undertaken in Lusaka urban clinics and our findings may not be generalizable to other settings. We note that six months follow-up time is a relatively short period and precludes a rigorous cost-effectiveness evaluation or assessment of the impact of integration on retention-in-care and long-term clinical outcomes for ART-enrolled patients. A cost effectiveness study will be necessary to provide information on the scalability of this model.

### Conclusion

The model presented here is one of the first case studies documenting the feasibility of formal integration of ART into general outpatient services at the primary health care level. Integration of care allowed shared use of space and staffing that resulted in increased HIV case finding, improved collection vital signs for OPD patients, a reduction in stigma associated with ART services but an overall increase in patient waiting times. This paper demonstrates ways in which resources for ART scale-up may be directed towards harmonizing service delivery systems in a high HIV-prevalence, low-resource setting. A rigorous evaluation is urgently required to assess true scalability, generalizability, long-term clinical outcomes and cost effectiveness.

## Supporting Information

Appendix S1 PITC Register, page 1.
(0.01 MB TIF)

Appendix S2 PITC Register, page 2.
(0.00 MB TIF)

Appendix S3 Pre-Implementation Time-in-Motion Study Form.
(0.01 MB TIF)

Appendix S4 Post-Implementation Time-in-Motion Study Form.
(0.22 MB TIF)

Appendix S5 Monthly OPD Attendances Clinics 1 & 2, 2006–2008.
(0.44 MB TIF)
