# Supplementary figures and images for: Strengthening Health Systems at Facility-Level: Feasibility of Integrating Antiretroviral Therapy into Primary Health Care Services in Lusaka, Zambia
Source: PLoS One. 2010 Jul 13;5(7):e11522. doi: 10.1371/journal.pone.0011522 (PMC2903482; doi:10.1371/journal.pone.0011522)

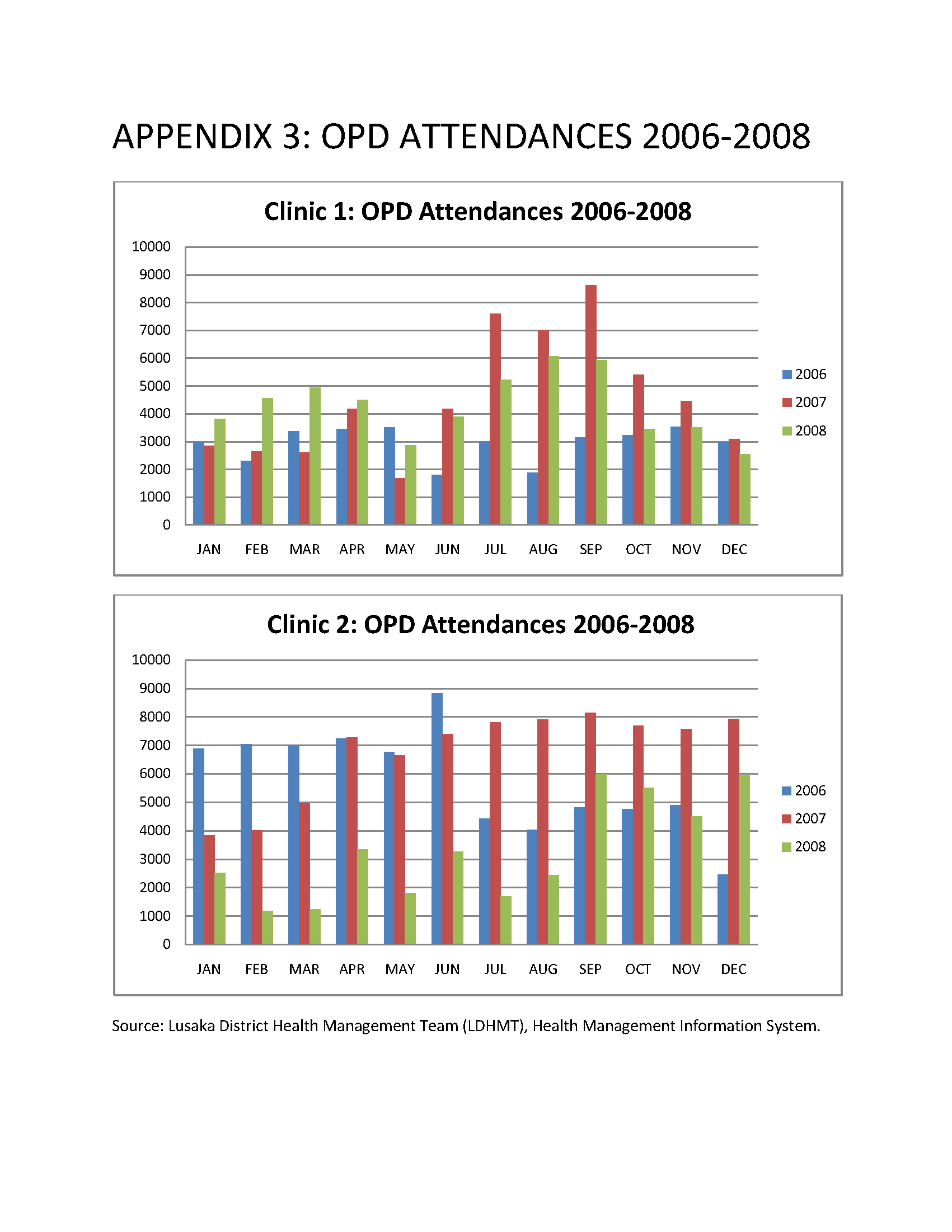

Supplement: Appendix S5 — Monthly OPD Attendances Clinics 1 & 2, 2006–2008. (0.44 MB TIF) [file pone.0011522.s005.tif]
